# "Opticians" and Sight Testing

**Published:** 1904-04-16

**Authors:** 


The Hospital.

A Journal of

The Medical Sciences and Hospital Administration.

Vol. XXXVI.—No. 916. APRIL 16, 1904.

### "Opticians" and Sight Testing.

A correspondence has lately been appearing in the columns of the *Times* newspaper which possesses considerable interest for the large number of persons who, at some period of their lives, require help from spectacles in the discharge of their daily duties, and which has related to the capacity of the ordinary spectacle seller, who calls himself an "optician," to decide what these spectacles ought to be, and to advise concerning the limits within which they should be employed. The designation "optician" is, in the great majority of cases, wholly misleading. Johnson defines the word to mean "one skilled in optics"; and optics is an abstruse science, the very elements of which require a considerable amount of mathematical knowledge. Sir Isaac Newton, or Thomas Young, or Sir George Stokes, might have been correctly described as an optician, and the application of the word to a "watchmaker and jeweller" in a country town, who could not read a mathematical formula if his life depended upon it, and whose claim rests, at the very most, only upon the circumstance that he buys spectacle lenses from one manufacturer and spectacle frames from another, and has learnt how to put them together, is a manifest absurdity. He displays in his window the legend "Spectacles to suit all sights," and he has been taught to supply an applicant of 45 or thereabouts, who asks for "clearers," with the weakest glasses through which type of a specified size can be read at a convenient distance. As a rule an applicant who is short-sighted is practically allowed to choose glasses for himself, or is told to take those through which he obtains the best distant vision, or, sometimes, those which fall a little short of producing this result. In either case a solemn warning may be given against glasses which are "too strong," and from the use of which, it is said, injurious results are to be expected. Forty years ago, before the work done by Professor Donders, of Utrecht, in investigating the conditions of sight, had become generally known, the spectacle seller of a large town, who carried on no other business except that of a trade in other optical instruments of a simple description, such as opera glasses and the like, was content to work upon the same lines as his provincial brother; but of late years, finding that glasses of various kinds, cylindrical, prismatic, and other, are being prescribed for patients by the medical profession, he has become fired with an ambition to do likewise, and to assume to himself the status of an authority upon the uses which should be made of his wares by those requiring them. In order to give some colour to his pretensions, he has been clamorous that the Worshipful Company of Spectacle Makers should examine him, and furnish him with something which would pass for a credential of competence; and he has now actually persuaded that Company to meet his views, and to issue what it is pleased to call a "diploma," on the strength of which he will probably hold himself forth as a competent and trustworthy adviser in all matters relating to the sight. In the newspaper correspondence he is very humble, and declares that all he requires is to be restrained within certain limitations by the Company. At present, as he puts the case, he has too much freedom with regard to that "right" of "testing vision" which has been exercised by spectacle sellers from the dawn of history, and he seeks to be prevented from doing mischief by being compelled to furnish some proof of knowledge and capacity.

The truth is that the whole contention of the spectacle seller is not only fallacious but preposterous. Every person with well-formed eyes begins to require spectacles for near work at about the age of 45, and, as the great majority of people have healthy eyes, so the great majority may safely take the glasses which give them comfortable sight at the required distance. The so-called optician may reasonably know that a power of a dioptre or thereabouts is usually the first requirement in such cases, and may be expected to supply it. He may also be expected to see to the proper fitting of the frame in which the glasses are held, and to know that upon this fitting much of the comfort of the user will depend. Beyond this it cannot be rendered safe or expedient for him to go, unless he has had a complete medical education. The eyes are not only optical instruments, they are living organs, and their purely optical defects are often connected with, or are even produced by, disease. In the emphatic language of Donders, for example, used as an expression of the results of between two and three thousand ophthalmoscopic examinations, "a short-sighted eye is a diseased eye;" and few cases require more careful weighing of medical and physiological conditions than those in which it is required to guide short-sighted eyes safely through the periods of study and of adolescence. The very numerous cases in which discomfort in using the eyes, or even incapacity to use them, is brought about by imperfect muscular co-ordination, and which call sometimes for optical and sometimes for surgical assistance, or for the former as a preparation for the latter, are wholly beyond the province of any but an experienced surgeon, equally conversant with all the methods by which relief may be afforded, and equally competent to apply whichever of them may be most suitable to the requirements of any given case. Even in what may appear to be simple presbyopia the risks of overlooking the beginnings of glaucoma, or the beginnings of nerve atrophy, or the beginnings of tobacco blindness, are by no means inconsiderable, and it is not to be expected that even the possession of a diploma will be a sufficient substitute for the "optician's" lack of previous physiological and medical training when he meets with these conditions. The moral of the whole case is summed up in the ancient apophthegm, *Ne supra crepidam sutor judicaret.* The scholar who devised the word "optologist" is requested to construe for "the trade."

